# Cerebrospinal fluid supports viability and proliferation of cortical cells *in vitro*, mirroring *in vivo *development

**DOI:** 10.1186/1743-8454-3-2

**Published:** 2006-03-20

**Authors:** Jaleel A Miyan, Mahjiub Zendah, Farhad Mashayekhi, P Jane Owen-Lynch

**Affiliations:** 1 Faculty of Life Sciences, The University of Manchester, 3.613 Stopford Building, Oxford Road, Manchester M13 9PT, UK; 2 Department of Biology, Faculty of Sciences, The University of Guilan, Iran; 3 Department of Biological Sciences, Lancaster University, Bailrigg, Lancaster, LA1 4YQ, UK

## Abstract

**Background:**

The central nervous system develops around a fluid filled compartment. Recently, attention has turned to the potential role of the fluid (cerebrospinal fluid, CSF) in the developmental process. In particular, the cerebral cortex develops from the germinal epithelium adjacent to the CSF with regulation of cell proliferation and differentiation provided by cells adjacent to the fluid-filled subarachnoid space.

**Methods:**

Histological analysis of fetal rat cortical sections was used to follow the extent of *in vivo *cortical development. A quantitative analysis of proliferation and migration of cortical cells at E17 – E21 was obtained through immunocytochemical staining of bromodeoxyuridine (BrdU) -labelled cells. *In vitro *studies were performed on primary cortical cells at days E17-E20, maintained in either Neurobasal media or 100% fetal rat CSF for 72 h before analysis of proliferation.

**Results:**

The proliferation potential of primary cortical cells varied depending on the age of extraction. E17 and E20 cells showed little proliferation while E18 and E19 cell showed the maximum. The CSF from fetuses of all ages tested, except E21, was able to maintain primary cortical cells from the developing fetus *in vitro *and to stimulate and support their proliferation in the absence of any additions. E17 cells showed little proliferation in any media while E19 cells showed maximum proliferation in E19 and E20 CSF.

**Conclusion:**

CSF composition most probably changes with age, as does the proliferation potential of cells in the developing cerebral cortex. CSF alone supports viability as well as proliferation of cortical cells. CSF must therefore be regarded as an important environmental influence in brain development and can be used *in vitro *to maintain both the viability of cortical progenitor cells and their age-related proliferative potential.

## Background

Using *in vitro *systems, the proliferation and differentiation of the stem and progenitor cells from the brain in response to added growth factors have been studied in some detail [1-5]. For example, these can be regulated by epidermal growth factor (EGF) [6,7], basic fibroblast growth factor (FGF2) [8,9], and transforming growth factor-beta (TGF-β) [10]. However, the influences acting on these stem and progenitor cells during development of the brain *in vivo *have not been characterised in any detail. It is likely that they are carried within the cerebrospinal fluid (CSF) [11] which contains large quantities of proteins including growth factors during development, in contrast to trace levels in adult CSF [12]. Historically, CSF studied in the context of the adult brain, has been associated with mechanical functions and some simple physiological functions, but it is becoming increasingly clear that the cerebrospinal fluid has a much more critical physiological role [13-15], particularly during development.

CSF is present from the early stages of neural tube development through to adult brain. In the early stages of brain development, cells within the ependymal lining of the neural tube are thought to secrete CSF. As the neural tube closes, a membrane formed from these cells invaginates to form the frond-like structures of the choroid plexus. This structure continues to secrete CSF throughout life. There is a developmental sequence in the appearance of the choroid plexus beginning with the 4th ventricle plexus followed by its appearance in the lateral and finally the third ventricles [16]. This sequence correlates with the later development of the cerebral cortex from germinal matrix cells, adjacent to the lateral ventricles. Thus a major component of the environment of germinal matrix stem and progenitor cells of the developing cortex, is the CSF system, known to contain growth factors secreted by the choroid plexus [17,18]. This may be particularly important in the period before vascularisation. However, it may be significant that the CSF remains adjacent to the germinal layer (and also the marginal zone) of the cortex throughout development and in later life.

Early experiments on chick embryos, involving CSF drainage, suggested that the CSF is important for the normal development of spinal cord and later development of the cortex [19,20]. In fact in cases where the flow of CSF is abnormal, for example through loss (e.g. spina bifida) or blockage (e.g. hydrocephalus), there is an associated abnormality in the development of the central nervous system (CNS), particularly of the cerebral cortex [11,15]. In the case of hydrocephalus, work with rat models of this condition points to a critical role of CSF in maintaining the normal proliferation of the stem cells in the developing cortex [13,14].

All these studies suggest that CSF is an important carrier of nutrients and factors involved in controlling and coordinating the progress of development of the CNS [11]. In this paper we have studied cortical cell proliferation *in vivo *using bromodeoxyuridine (BrdU) staining of tissue sections and have used *in vitro *assays to assess the ability of CSF to support cortical cell proliferation, focusing on the critical stages of development.

## Materials and methods

### Animals

All experiments were sanctioned by the Home Office Animal Procedures Inspectorate. The Wistar rat colony was started from breeding pairs purchased from Charles River (UK). These were maintained as non-SPF on a 12:12 h light dark cycle beginning at 8.00 am. They were kept at a constant temperature with unrestricted access to food and water. The colony was maintained through random pair mating. Timed mating was carried out by placing a male and female together and checking for the presence of a vaginal plug every 4 h. The presence of a plug was taken to indicate successful mating and the time taken as gestational day zero, E0. Fetuses from timed pregnancies of Wistar rats were harvested after euthanasia of the mother, by intraperitoneal injection of an overdose of anaesthetic (sodium pentobarbitone), at gestation days, E17-E20 and brains were removed and processed as described below.

### Staining and immunocytochemical analysis of tissue sections

For histological analysis, brains were fixed in 4% paraformaldehyde for a minimum of 2 h, cryopreserved in 20% sucrose, and frozen in iso-pentane cooled with dry ice. Serial coronal sections, 20μm-thick, were obtained with a Leica CM cryostat and stained with haematoxylin and eosin.

To identify proliferating cells, 5-bromo-2'-deoxyuridine (BrdU, Sigma, Poole, UK) was administered to pregnant dams by intraperitoneal injection at a dosage of 60 mg/kg at E17. Fetuses were then recovered at E18-E21, after BrdU injection of their dams, and the heads fixed in ice cold 4% paraformaldehyde in phosphate buffered saline (PBS) at pH 7.3. Fixation was carried out for a minimum of 2 h and usually overnight. Coronal sections were incubated in 10% normal serum in PBS for 2 h prior to flooding with primary antibody, a mouse anti-BrdU antibody (Nova Castro Laboratories, Newcastle UK), diluted 1:1000 in 10% normal serum in PBS, overnight at 4°C. Sections were then washed in PBS (3 × 1 h) and flooded with secondary antibody, universal biotinylated horse anti-rabbit-anti-mouse secondary antibody (Vector Laboratories Ltd, Peterborough UK), at a dilution of 1:200 in 10% normal serum in PBS for 2–4 h. Visualisation was by avidin-biotin-peroxidase diaminobenzidine staining (Vector Elite ABC Kits). All stained sections were mounted in glycerin-albumin and images were captured using a Coolsnap digital camera (Princeton Instruments) fitted to a Leica DMLB microscope. Image-capture and analysis was carried out using Metaview software (Universal Imaging Corporation). Different regions of the coronal cortical section, the germinal matrix, the intermediate zone and the cortical plate, were identified and the number of positive cells in each zone was counted on a photomontages constructed from micrographs taken at x400 magnification across the cortex. These gave a strip of cortex 400μm wide from the ventricular ependymal layer to the surface of the cortex next to the pia.

In all experiments, a minimum of three measurements (BrdU positive cell counts in the region of the cortex under analysis) was taken for each individual to give an average for each zone for that individual. These averages from at least four separate fetuses from separate litters were pooled to give a mean ± SEM.

### Preparation of cell cultures

Cortical hemispheres from the brains of embryonic rats at E17-E20 were used to prepare primary cell cultures. 4–6 hemispheres were cleared of meninges and incubated in trypsin-EDTA solution (0.25%) at 37°C for 20 min. The solution was replaced with Neurobasal medium (Invitrogen, Paisley, UK) and the cells dissociated by repetitive pipetting through tips of decreasing bore size. The suspension was centrifuged at 1700 rpm for 5 min and the supernatant replaced with fresh media. Further pipetting was performed to break up clusters of cells before a sample was taken for viability staining with trypan blue (0.4%) and cell counting. The dissociated cells were plated in poly-D-lysine (0.05 mg/ml) coated 96 well plates at a density of 1 × 10^5 ^cells/ml in Neurobasal medium which preferentially supports progenitor and neuronal cell types (not glial cells), containing B27 supplement (Invitrogen), 2 mM glutamine and penicillin-streptomycin (0.1 mg/ml). At 24 h the media was replaced with 50 μl fresh Neurobasal medium (media only) or, alternatively, cells were incubated in 50 μl 100% CSF from different age sources (as indicated in the figure legends) and the cultures were maintained at 37°C in a 5 % CO_2 _atmosphere for a further 48 h.

### Proliferation assay

Cell proliferation was determined using the luminescence-based Lumitech Vialight (LumiTech Ltd, Nottingham UK) high sensitivity cell proliferation and cytotoxicity assay kit (as per manufacturers instructions), which is based on the measurement of concentrations of ATP in the culture. Analysis of the luminescence reading obtained against known viable cell numbers for cortical cells from fetuses showed that the assay is linear for cell numbers up to 1 million cells/well. This assay can detect as few as 100 viable cells in the culture and thus is highly sensitive in comparison with other methods for determining the number of cells within a culture. It measures cells that have actually completed division, unlike alternative methods, which measure incorporation of nucleotides into DNA. Measurements were made on a Multilabel Counter (Wallac Victor2 1420, PerkinElmer, UK). Luminescence measurements were taken from control wells plated at the initiation of the culture and assayed immediately. These give control day zero luminescence readings. Results are expressed as fold of this day zero measurement, mean +/- SEM of at least three experiments involving at least three litters, each performed in triplicate.

### Collection of CSF samples

In experiments to determine the effect of CSF on cortical cell cultures, CSF was removed by tapping the cisterna magna of Wistar rat fetuses immediately after extraction from the dams as described above. This CSF was added to the cultures as shown. The cisterna magna is an excellent site to collect uncontaminated CSF since the fluid space is large and access is through a thin membrane once the overlying musculature has been dissected. Occasional contamination with blood was caused by severing a blood vessel within the cisternal cavity and these samples were discarded. All samples were collected into sterile Eppendorf tubes and routinely centrifuged twice at 14,000 rpm to remove any cells or debris from the fluid, which was decanted into another sterile tube. Samples were frozen on dry ice and stored at -80°C until use. Between 5 and 50μl of CSF was collected from each fetus using this method and CSF from individual litters was pooled for the experiments.

## Results

### Analysis of the changes in cortical morphology in development

**Figure 1:**
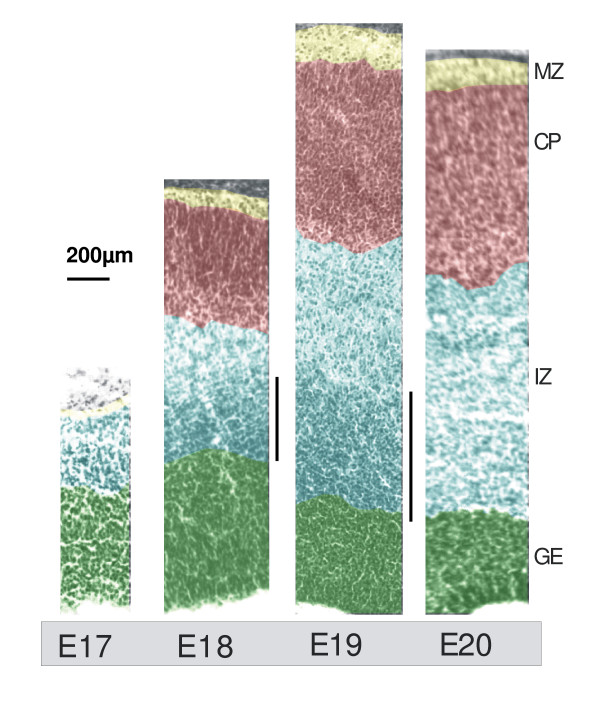
Development of the cortical zones between E17–20. False-coloured photomontages of H&E stained coronal sections across the thickness of the cortical mantle from the brains of E17-E20 Wistar rat pups with the ventricular surface at the lower edge. Montages were selected as representative specimens from at least 3 separate pups from different litters. At E17 there is no recognizable cortical plate while at E18 it is well developed. At E18 and E19 there is a zone above the GE (marked with a vertical line) that may represent a concentration of cells produced from the proliferation occurring in the GE, which have yet to migrate. The GE decreases and the IZ and CP increase in thickness with age. GE: Germinal epithelium; IZ: intermediate zone; CP cortical plate; MZ: marginal zone.

The rat cortex (or more accurately the cortical plate) appears over days E17-E21. In order to examine this development in more detail, we analysed photomicrographs of sections through the cortex across this time scale. There was some increase in total thickness from E17 to E20 and a rapid appearance of cortical plate over the first 48 h (Fig. 1), demonstrating that this is a critical period in the generation and migration of the neurones of the cortex from the germinal epithelium through the intermediate zone into the cortical plate. By E19 the cortical plate was well established and the thickness of the germinal epithelium appeared to decline as the mass of migrating neurones left the intermediate zone (Fig. 1).

**Figure 2:**
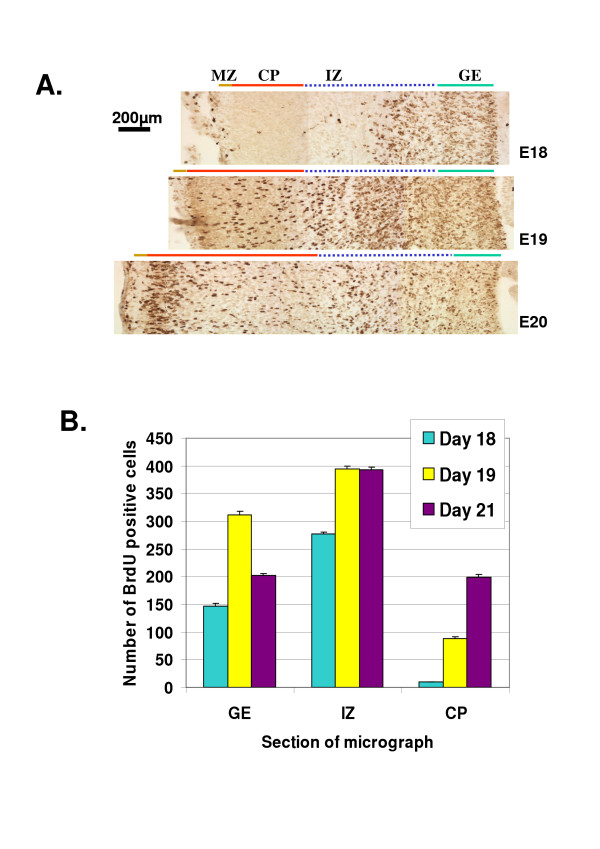
Proliferation within cortical zones over E18–20. BrdU was administered to pregnant dams at E17. Fetuses were recovered at E18 – E21 and coronal sections were stained as described. (A) Photomontages of representative cortical sections stained with antibody to BrdU showing the progression with age of cells born on E17 from the germinal epithelium into the intermediate zone and cortical plate by E18 – E20. MZ (brown bar): marginal zone. (B) The number of BrdU positive cells, counted on 400 μm-wide ventricle-to-pia sections, in the germinal epithelium (GE, green bar), intermediate zone (IZ, blue dashed bar) and cortical plate (CP, red bar) at days E18, E19 and E21. Results for each age are from 4 separate experiments. Data are means ± SEM.

In order to assess patterns of germinal epithelium cell proliferation and migration *in vivo*, BrdU was injected into pregnant dams at E17 to analyse cell generation and location over the subsequent 2–4 days. Cells generated on E17 were present in the germinal matrix and intermediate zone on E18, began to move into the cortical plate by E19, and were more concentrated in the cortical plate by E20 and E21 (Figs. 2A and 2B).

### Proliferation of primary cortical cells in Neurobasal medium

**Figure 3:**
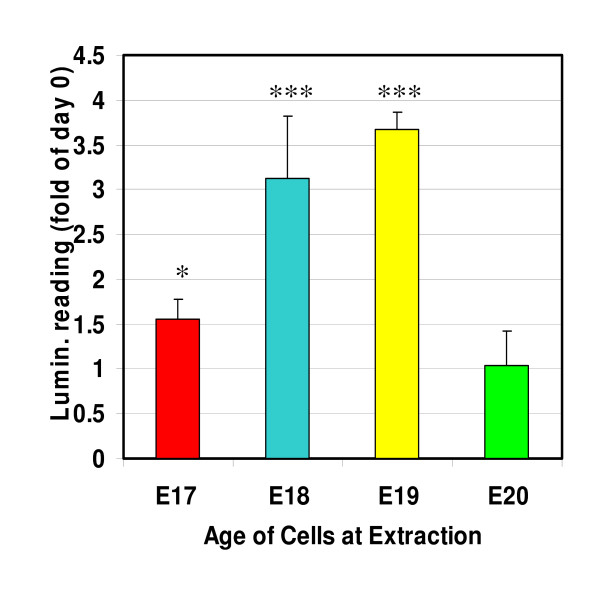
*In vitro *proliferation of cortical cells extracted from E17–20. Cortical cells were incubated in supplemented Neurobasal medium. Data are presented as fold of control (day 0 luminescence value) means ± S.E.M. of a minimum of five experiments each performed in triplicate. *** *P *< 0.001, *P < 0.05 compared to day 0 as determined by *t*-test analysis.

Cortical germinal epithelial (GE) cells are usually maintained *in vitro *in a specialised Neurobasal medium containing B27 supplement (Invitrogen, Paisley UK), which preferentially supports the growth of progenitor and neuronal cell types by enhanced proliferation and self-renewal [2,5,21]. Histological analysis as shown in Fig. 1, suggests that it is important to consider the exact age of the cells at extraction, since the apparent rate of proliferation changes over a single day, implying that the response profile of the primary cells derived from these time points would be different. The growth of E17 – E20 cells (the days known to be critical for cortical development in the embryonic rat) in Neurobasal media is compared in Fig. 3. There was a time window over days E18 and E19 of gestation where the cells exhibited a large significant, proliferative response (*P *< 0.001 compared to time zero sample as analysed by Students *t *test). In contrast on either side of this time window, on days E17 and E20, the proliferation of the cells under these conditions was minimal and only significant at E17 (*P *< 0.05).

### Effects of CSF from different gestational ages on viability and proliferation

The above experiments show that there was an age-related variation in the ability of primary cortical cells to proliferate and that this has at least two important components, an age-related proliferation potential presumably dictated by developmental genes, and their *in vivo *environment which provides the support required for growth, proliferation, differentiation and migration.

**Figure 4:**
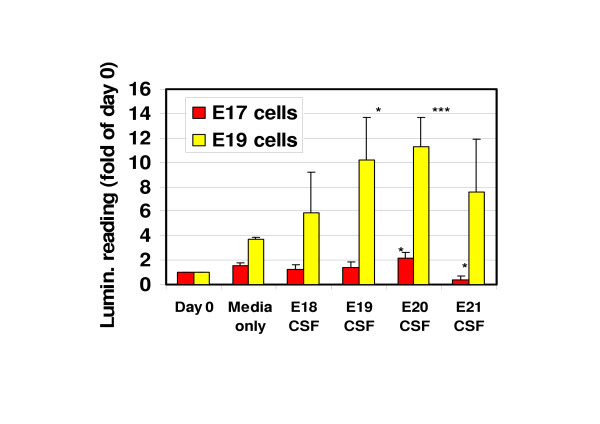
Effect of CSF on the in vitro proliferation of cortical cells. Cortical cell cultures from E17 (red) or E19 (yellow) Wistar rat brains were incubated in either supplemented Neurobasal medium (control) or 100% CSF from E18, E19, E20 or E21 fetal brains and proliferation assessed by luminescence assay. The luminescence of control cell cultures at the point of plating out the assays was also measured. Data are presented as fold of control (day 0 luminescence value). Data are means ± S.E.M. of a minimum of three experiments each performed in triplicate. *** *P *< 0.001, *P < 0.05 compared to day 0 as determined by *t*-test analysis.

Based on previous work pointing to a role for CSF in cortical development, we examined whether the cultured cortical cells from different fetal stages could be maintained in CSF alone. Primary cortical cells were taken from E17 and E19 fetuses whilst CSF was collected from fetal brains aged E18 through to E21. The medium was completely replaced by CSF such that cells were incubated in 100% CSF. In all cases CSF was able to maintain the E19 cells in a viable state (Fig. 4). In fact not only was cell viability maintained, but cells also proliferated in the CSF to an extent comparable to, or, in excess of, that observed with normal Neurobasal medium (Fig. 4). The response of the E17 cells was significantly reduced compared to the E19 cells but even with these cells, the CSF was able to maintain cell viability over the 72 h period. The only exception to this was E21 CSF, which was unable to support the survival of E17 cells, a perhaps not unexpected result given the temporal separation between these two components of the developing cortex *in vivo*.

Together these data illustrate that CSF alone is able to function as a medium for survival and proliferation of developing cortical cells *in vitro *and that CSF composition changes with time as reflected in its changing ability to support viability and proliferation.

## Discussion

The data presented here show that the *in vitro *proliferative potential of primary cells derived from the developing cerebral cortex of rats was dependent upon the age of extraction. As well as highlighting the necessity for careful age matching of cell samples taken from the developing cortex, these experiments also pointed to the ability of the CSF to function as a physiological medium for the growth of germinal epithelium stem and progenitor cells. The CSF was able to maintain viability of the cortical epithelial cells and stimulate their proliferation. In fact, given that at the early stages of development the cortical epithelium contains only germinal matrix stem cells, it is highly likely that the CSF can maintain the viability and induce proliferation of these brain stem cells in *in vitro *cultures.

In terms of its role *in vivo*, CSF is present from the earliest stage of neural tube development when cells within the ependymal lining of the neural tube are thought to secrete the fluid [22]. As the neural tube closes, the choroid plexus is formed and this structure then continues to secrete CSF throughout life. In addition to the secreted CSF, there are significant contributions of fluid and components from the bulk flow of interstitial fluid from the brain parenchyma thought to be up to 15–20% of the total volume of CSF. Thus a major component of the environment of germinal matrix stem and progenitor cells of the developing cortex is the CSF and, as shown here, this fluid can support the viability and proliferative potential of these cells as they pass through the developmental programme associated with formation of the cerebral cortex.

Since many of the growth factors important to development of the cortex are known to be secreted by the choroid plexus directly into the CSF [17,18], it is not unreasonable to conclude that the CSF is vital to the developmental process. Of course, the cells *in vivo *will experience additional influences through contact with other cells and locally secreted factors, most notably from glial cells. Further support for the critical role of CSF comes from the recent work by Gato and colleagues [23]. They demonstrated that slices of developing chick brain were incapable of supporting neurogenesis or migration of neurones from the germinal epithelium unless CSF was added to the growth medium. This suggests that, rather than the critical growth factors being produced in the local microenvironment of the brain, the surrounding CSF is sufficient to drive the developmental process.

Another aspect of CSF function is its flow pathway. Cortical development coincides with the opening of the foramen of Luschka and Magendie at E17 and the consequent flow of CSF from the ventricular system into the subarachnoid spaces [11]. This may be related to a need for signals (e.g. BDNF) [24,25] to reach the pial meningeal cells to initiate and promote reelin output from Cajal-Retzius cells in the marginal zone. During this critical period of development Saunders and colleagues [26,27] have shown the presence of an ependymal barrier to free movement of fluid through to the brain parenchyma from the CSF, in the later stages of gestation from E16 through to birth. This ensures that the CSF only reaches the pial meningeal cells after the foramina of Luschka and Magendie open [28]. There is a strong correlation between this opening of the foramina and triggering of normal migration of neurones and stratification of the cortex [29-34]. In spina bifida aperta, where CSF can fail to enter the subarachnoid space, cortical development is severely affected [15]. In early-onset hydrocephalus, where CSF flow is obstructed at this critical period of cortical development, cortical development is also severely affected [13,14].

## Conclusion

The data presented here along with the evidence from the studies discussed above, implicates the CSF as an important element in the development of the central nervous system, and specifically of the cerebral cortex.

## Competing interests

The author(s) declare that they have no competing interests.

## Authors' contributions

PJOL conceived and designed the study, analysed and interpreted all the data and drafted/re-wrote the manuscript. MZ and FM acquired the data and did some preliminary analysis. JAM also conceived the study and drafted/re-wrote the manuscript. All authors have read and approved the final version of the manuscript.
